# Enhancing grid-connected photovoltaic system performance with novel hybrid MPPT technique in variable atmospheric conditions

**DOI:** 10.1038/s41598-024-59024-4

**Published:** 2024-04-08

**Authors:** Layachi Zaghba, Abdelhalim Borni, Messaouda Khennane Benbitour, Amor Fezzani, Abdullah Alwabli, Mohit Bajaj, Shir Ahmad Dost Mohammadi, Sherif S. M. Ghoneim

**Affiliations:** 1 Centre de Développement des Energies Renouvelables, CDER, Unité de Recherche Appliquée en Energies Renouvelables, URAER, 47133 Ghardaïa, Algeria; 2 Department of Electrical Engineering, College of Engineering and Computing in Al-Qunfudhah, Umm Al-Qura University, Mecca, Saudi Arabia; 3 Department of Electrical Engineering, Graphic Era (Deemed to Be University), Dehradun, 248002 India; 4 Hourani Center for Applied Scientific Research, Al-Ahliyya Amman University, Amman, Jordan; 5 Graphic Era Hill University, Dehradun, 248002 India; 6 Applied Science Research Center, Applied Science Private University, Amman, 11937 Jordan; 7 Department of Electrical and Electronics, Faculty of Engineering, Alberoni University, Kapisa, Afghanistan; 8 Department of Electrical Engineering, College of Engineering, Taif University, 21944 Taif, Saudi Arabia

**Keywords:** PV plants, Hybrid MPPT, PSO, GA, Atmospheric conditions, Energy science and technology, Engineering, Mathematics and computing

## Abstract

This paper proposes an innovative approach to improve the performance of grid-connected photovoltaic (PV) systems operating in environments with variable atmospheric conditions. The dynamic nature of atmospheric parameters poses challenges for traditional control methods, leading to reduced PV system efficiency and reliability. To address this issue, we introduce a novel integration of fuzzy logic and sliding mode control methodologies. Fuzzy logic enables the PV system to effectively handle imprecise and uncertain atmospheric data, allowing for decision-making based on qualitative inputs and expert knowledge. Sliding mode control, known for its robustness against disturbances and uncertainties, ensures stability and responsiveness under varying atmospheric conditions. Through the integration of these methodologies, our proposed approach offers a comprehensive solution to the complexities posed by real-world atmospheric dynamics. We anticipate applications in grid-connected PV systems across various geographical locations and climates. By harnessing the synergistic benefits of fuzzy logic and sliding mode control, this approach promises to significantly enhance the performance and reliability of grid-connected PV systems in the presence of variable atmospheric conditions. On the grid side, both PSO (Particle Swarm Optimization) and GA (Genetic Algorithm) algorithms were employed to tune the current controller of the PI (Proportional-Integral) current controller (inverter control). Simulation results, conducted using MATLAB Simulink, demonstrate the effectiveness of the proposed hybrid MPPT technique in optimizing the performance of the PV system. The technique exhibits superior tracking efficiency, achieving a convergence time of 0.06 s and an efficiency of 99.86%, and less oscillation than the classical methods. The comparison with other MPPT techniques highlights the advantages of the proposed approach, including higher tracking efficiency and faster response times. The simulation outcomes are analyzed and demonstrate the effectiveness of the proposed control strategies on both sides (the PV array and the grid side). Both PSO and GA offer effective methods for tuning the parameters of a PI current controller. According to considered IEEE standards for low-voltage networks, the total current harmonic distortion values (THD) obtained are considerably high (8.33% and 10.63%, using the PSO and GA algorithms, respectively). Comparative analyses with traditional MPPT methods demonstrate the superior performance of the hybrid approach in terms of tracking efficiency, stability, and rapid response to dynamic changes.

## Introduction

PV plants are environmentally friendly, safe, and reliable sources of energy, they have played an essential role in renewable energy technologies^1^. PV-based renewable energy solutions have attracted considerable interest for both grid-tied and independent installations^2,3^. The international market for solar electricity is currently growing at an average rate of 30–40% per year for over ten years^4^. This unprecedented increase, mostly owing to solar systems connected to the energy distribution network, has resulted in technological improvements and decreased PV module costs, as well as substantial initiatives for research and development in the area associated with power^5^. In fact, the PV sources and dependability of converter used to link solar systems to power distribution network modules are specifications that can significantly affect the yearly output of energy and, as a result, the economic sustainability of a the plant^6,7^. One of the goals of solar systems linked to the grid is to manage the current and power supplied into the network in accordance with international standards^8^. Therefore, specifications that have historically used to design an inverter are the rated power, the rated voltage of the network, maximum DC link voltage, the inverter command, etc. Some aspects can make significant improvements to the design and practical implementation of the DC–AC converter linked to the conventional network, namely the control of the power factor, reducing harmonic distortion, using the command digital to eliminate the DC component of the current supplied to the grid, etc.^9,10^. Another very important aspect of photovoltaic installations that are grid-connected is the type of energy supplied into the network, whether reactive or active, which can change the type of power factor^11,12^. The most efficient systems are those that can vary the power according to grid requirements. External elements such as temperature and solar radiation have an impact on solar PV systems^13^. As a result, for better efficiency, the PV array should continually operate at the extreme power point (MPPT). In this situation, the MPPT controller is a critical component that must be included in every solar PV system in order to assure a high generating capacity^14,15^.

Several studies in the literature investigated different kinds of MPPT control strategies. These approaches are chosen according to their requirements, such as simple implementation, accuracy, cost effectiveness, and time calculation^16^. Perturb and Observe^17^ and incremental conductance^18^, are the two most frequently employed methods because they are easy and simple to use. Nevertheless, they have difficulties in that they are unable to follow the MPP during the fast of sunlight fluctuations. Furthermore, they exhibit fluctuation toward the maximum power point under standard operating conditions and low convergence when temperature and/or irradiance vary rapidly. A lot of work has been published on this topic in the last year. Employing FS-MPC controllers linked to the grid, a novel solar power plant strategy based on metaheuristic algorithms is given in^19^. The PSO and finite set model predictive controller (FS-MPC) are compared with conventional P&O. The findings reveal rapid convergence and a few oscillations. Authors describe how to optimize a fuzzy-based-MPPT approach for standalone PV systems using meta-heuristic methods. When the suggested techniques are compared to traditional P&O, the results indicate good performance in low and high irradiation conditions and verify performance in transient conditions^20^. Authors show how ANFIS MPPT management and twin-axis sun monitoring may improve the performance of solar energy systems linked to the network. The simulation outcomes show that the neuro-fuzzy approach performs well not only in terms of pursuing the highest power point but also in terms of adaptability, speed, and output precision^21^. Authors introduce a genetic algorithm (GA)-based upgraded P&O-PI MPPT controller for stationary and twin-axis tracking grid-linked solar systems. Greater performance is suggested by the simulation findings. This approach can swiftly increase the quantity of energy harvested and their efficiency^22^. Authors illustrate a smart PSO-Fuzzy MPPT approach for solar power plants in extreme climates. PSO is employed to determine the optimal gains. When compared to the traditional algorithm, the results demonstrate quicker monitoring of power maxima with minimal oscillation and a shorter reaction time^23^. In^24^, improved MPPT controllers using GA for network-linked solar plants are presented. A comparison of P&O-PI and fuzzy-PI MPPT algorithms optimized with a genetic algorithm (GA) is presented. The findings reveal that the fuzzy logic-PI MPPT algorithm outperforms the P&O-PI controller. In^25^, a comparison of fuzzy-PI MPPT and P&O-PI techniques optimized using PSO and GA for grid-connected solar plants is made. The results demonstrate that the fuzzy-PI controller with PSO algorithms is preferable to other methods about performance specifications. Boost converter real-time evaluation using a MyRio controller and hybrid neural network PSO-RBF approaches was presented by^26^. PSO is used to forecast and minimize the RMSE error. The results demonstrate quick convergence, excellent efficiency, and adequate performance under partial shading. Authors created a combination of an ABC and ANFIS- -based MPPT operator for solar power plants with anti-islanding network security. The ANFIS membership function has been adjusted by the artificial bee colony. The findings suggest that the approach is highly effective^27^. Authors provide an optimized P&O algorithm together with an artificial bee colony for solar plants that operate in partial shade. The ABC determines the GMPP, and P&O are used to determine it more precisely. The findings demonstrate that the suggested approach prevents fluctuations at the steady state, is suitable for partial shading, has a short payback period, and is very efficient^28^. Authors employ a novel combination of BAT-fuzzy controller-based MPPT for grid-tied PV-battery plants. The bat will find the ideal controller value, and the Fuzzy is going to optimize the bat. The results demonstrate quick convergence, excellent efficiency, and adequate performance under partial shading^29^. Authors propose an innovative MPPT approach based on HGO optimization. The findings of HGO are compared to those of other algorithms such as PSO, DFOA, CSA, and GHO. The HGO reduces settlement period, minimizes tracking time and enhances efficiency^30^. In^31^, a novel HHO MPPT approach is used to successfully monitor the golabla MPPT under all situations. The suggested HHO has higher performance in the MPP and their rapid convergence. A novel MPPT approach based on Salp Swarm Optimization (SSO) is suggested by^32^. The results of SSO are compared to those of various MPPT approaches such as PSO, DFO, CS, ABC, and PSOGS. SSO enhances tracking and stability. An analytical investigation validates the suggested SSO’s robustness and sensitivity. A robust global MPPT of a solar cell-based plant utilizing the MRFO algorithm is demonstrated in^33^. The acquired results are compared to those produced using both the DE and the CSA algorithms. The collected findings validated the suggested MPPT’s efficiency under various partial shadow patterns. In recent years, several important recent studies regarding intelligent control techniques, such as neural networks^34,35^, fuzzy logic^36,37^, particularly those relying on GMPPT approaches, such as particle swarm optimization (PSO), have demonstrated remarkable stability in the face of unpredictable climatic fluctuations. The literature review highlights the persistent challenge faced by traditional MPPT systems in efficiently extracting energy from PV fields under varying environmental conditions. Despite advancements in MPPT techniques, existing approaches often struggle to adapt to fluctuations in sunlight intensity and ambient temperature, leading to suboptimal performance and reduced energy yield^38^. The identified research gap underscores the need for a novel MPPT technique that can overcome the limitations of traditional systems and enhance the overall performance of grid-connected PV systems. Specifically, such a technique should be capable of accurately tracking the maximum power point (MPP) of PV arrays under real-world atmospheric conditions characterized by dynamic sunlight and temperature profiles.

This paper presents a pioneering contribution in the field of photovoltaic (PV) systems by introducing a novel hybrid Maximum Power Point Tracking (MPPT) technique that integrates fuzzy logic and sliding mode control to obtain better tracking performance (DC side). The sliding mode technique has several significant advantages: high precision, stability, simplicity, invariance, and robustness. Unfortunately, the sign function causes chattering on the sliding surface, which is normally unfavorable. In this paper, we propose subtitling the function sign by the fuzzy logic function. The second contribution of this research is focused on adjusting the PI gains of the control loop of inverter utilizing the Genetic Algorithm (GA) and PSO approaches (AC side). The proposed approach is designed to optimize the performance of grid-connected PV systems under real-world conditions characterized by variable solar irradiation and ambient temperature. By combining the adaptive capabilities of fuzzy logic with the robustness and fast response of sliding mode control, the proposed technique aims to enhance the efficiency of energy extraction from PV arrays. Furthermore, advanced optimization methods such as Particle Swarm Optimization (PSO) and Genetic Algorithm (GA) are employed to fine-tune the parameters of the MPPT controller, ensuring optimal tracking efficiency and stability. Through extensive simulation studies, the effectiveness of the hybrid MPPT technique is demonstrated, showcasing its ability to maximize energy yield, improve response time, and maintain stability even in dynamically changing environmental conditions. A comparative analysis with existing MPPT methods underscores the superior performance and effectiveness of the proposed approach, thereby contributing significantly to the advancement of MPPT techniques for grid-connected PV systems.

The work is structured as bellow: section “Collection of data” covers the system, including the solar panel parameters, converter characteristics, and the suggested MPPT approach. Section “Description of the grid-connected PV system”: simulation findings. Finally, section “Simulation results” concludes the article.

## Collection of data

**Figure 1:**
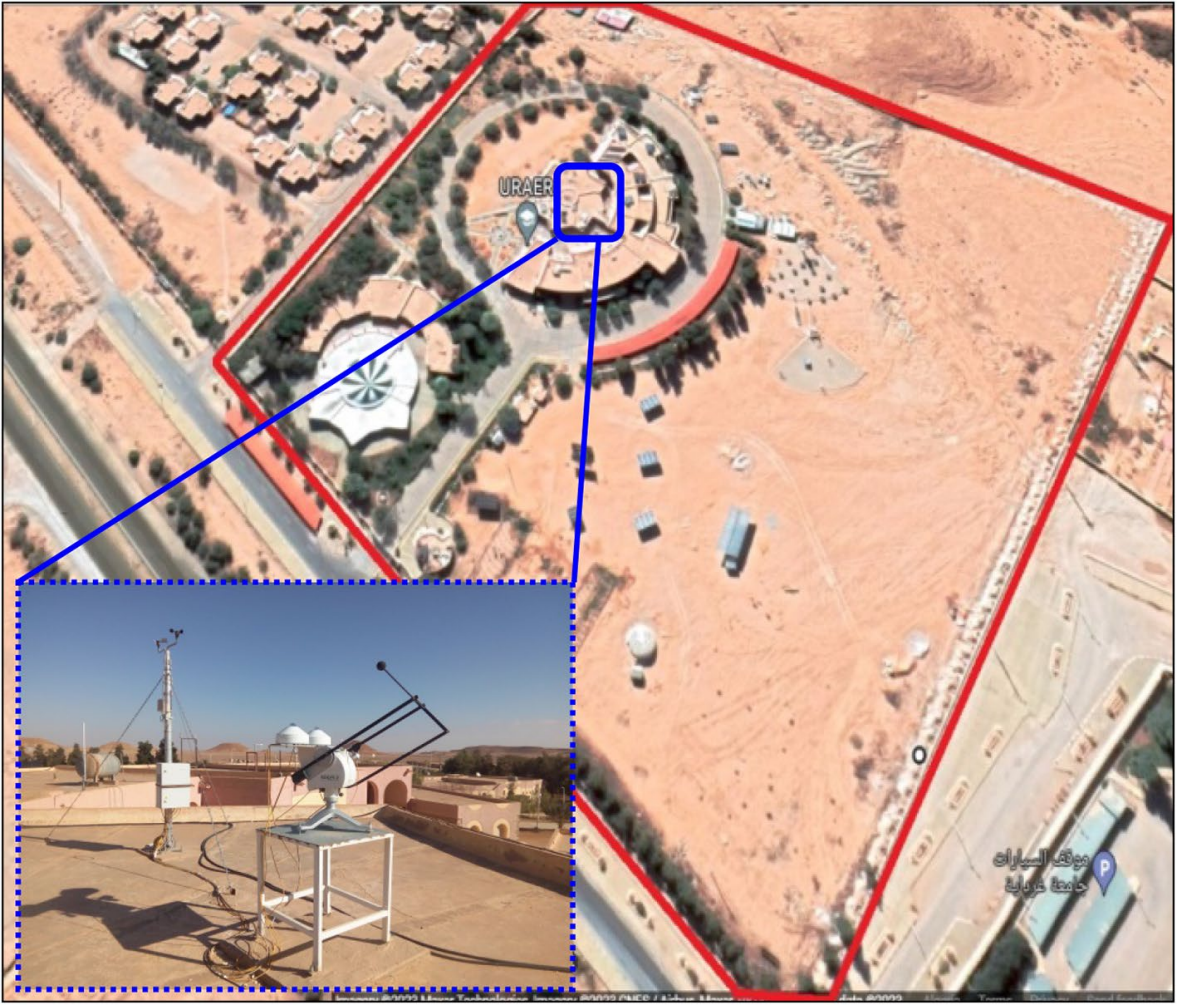
Photograph of meteorological station implanted in URAER Ghardaia.

The meteorological data (solar radiation and temperature) were taken each 5 min with great accuracy using a CM11 Pyranometer type installed on the URAER’s rooftop, as illustrated in Fig. 1.

**Figure 2:**
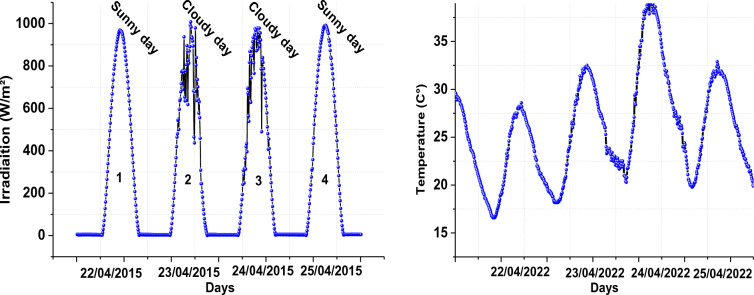
Global solar radiation and ambient temperature profile within 4 days.

A Fig. 2 shows the radiation and ambient temperature that were measured between April 22 and April 25, 2015.

## Description of the grid-connected PV system

**Figure 3:**
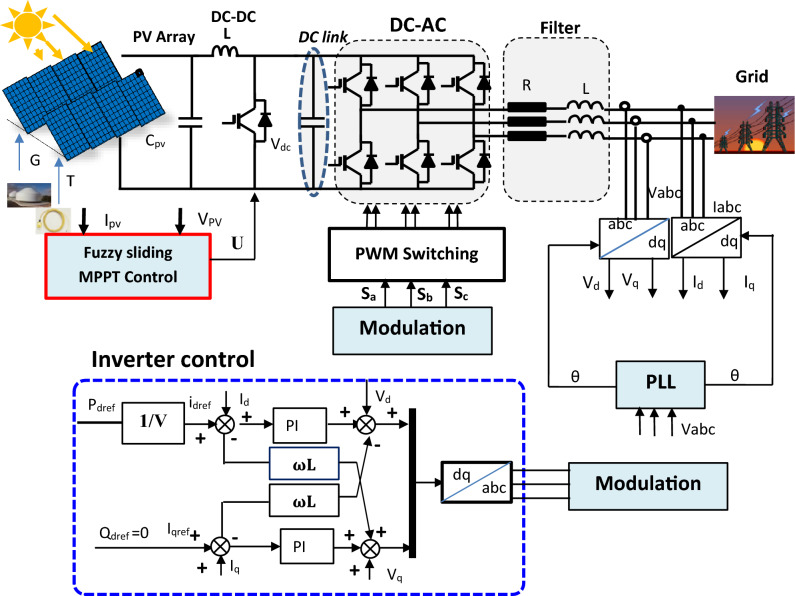
Block schematic of a grid-linked PV system.

Grid-linked photovoltaic (PV) plant is a solar power system that is connected to the electrical grid^39,40^. It consists of solar panels, an inverter, and a connection to the utility grid (see Fig. 3).

### PV side control

#### PV array modeling

**Figure 4:**
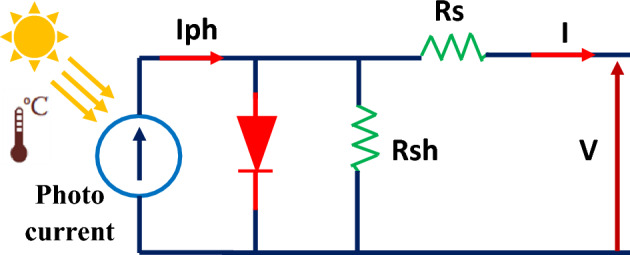
Equivalent circuit of a photovoltaic array.

Modeling the equivalent circuit of a photovoltaic (PV) cell is essential for understanding its behavior and for designing efficient PV systems^41,42^. The most commonly used equivalent circuit model for a PV cell is the single diode model. This model represents the PV cell as an ideal current source in parallel with a diode and a resistor (Fig. 4). The equations for the single diode model are as follows^34,35^:

1 $$ \left\{ {\begin{array}{*{20}l} {I = Iph - Is\left( {exp\left( {\frac{{q\left( {V + RsI} \right)}}{{N_{s} KTa}}} \right) - 1} \right) - \frac{V + RsI}{{Rsh}}} \hfill \\ {I_{pv} = \frac{G}{{G_{r} }}\left[ {I_{pvn} + K_{1} \left( {T - T_{r} } \right)} \right.} \hfill \\ {I_{s} = \frac{{I_{scn} + K_{1} \left( {T - T_{r} } \right)}}{{{\text{exp}}\left( {V_{ocr} + K_{V} \left( {T - T_{r} } \right)/a(N_{s} KT} \right) - 1)}}} \hfill \\ \end{array} } \right. $$

The output current of PV array is given by the following expression.

2 $$ I = I_{ph} - I_{0} N_{pp} \left[ {exp\left( {\frac{{V + R_{s} \left( {\frac{{N_{ss} }}{{N_{pp} }}} \right)I}}{{V_{T} aN_{ss} }} - 1} \right)} \right] - \frac{{V + R_{s} \left( {\frac{{N_{ss} }}{{N_{pp} }}} \right)I}}{{R_{sh} \left( {\frac{{N_{ss} }}{{N_{pp} }}} \right)}} $$

where $I$, ${I}_{ph}$ and ${I}_{0}$ are the current array, the photo generated, and the reverse saturation current, respectively. $V$, ${V}_{T}$ are the array voltage and the thermal, respectively. $a$ is the diode ideality factor for the single diode model. ${R}_{s}$, ${R}_{sh}$ are cell series and shunt resistance. ${N}_{ss}$, ${N}_{pp}$ are the number of modules in series and parallel. $q$ is the electron charge [1.60217646 * 10–19 C]. $k$ is the Boltzmann constant [1.3806503 * 10–23 J/K].

#### Fuzzy sliding MPPT approach with Boost converter model

**Figure 5:**
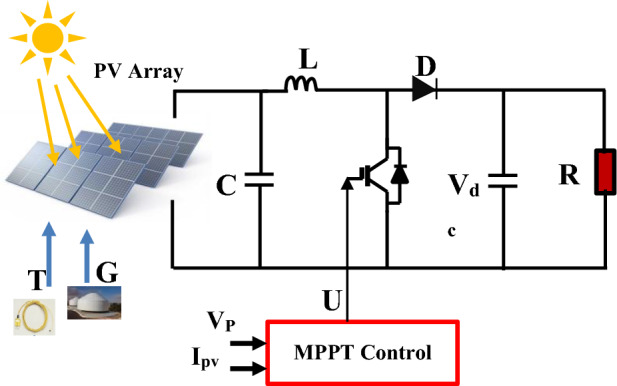
A solar generator coupled to a DC-DC boost converter.

Figure 5 depicts the schematic representation of a static boost converter coupled to a solar generator^36,37^.

#### Control of the sliding mode

The sliding technique has various significant benefits, including high accuracy, strong stability, simplicity, invariance, resilience, etc.… We may achieve this based on the solar array’s features while it is running at its maximum output power condition^43,44^.

3 $$ S = \frac{{\partial P_{PV} }}{{\partial V_{PV} }} = \frac{{\partial (V_{PV} \cdot i_{PV} )}}{{\partial V_{PV} }} = \frac{{\partial i_{PV} }}{{\partial V_{PV} }}V_{PV} + i_{PV} = 0 $$

4 $$ U = \left\{ {\begin{array}{*{20}l} 0 \hfill & {\quad S \ge 0} \hfill \\ 1 \hfill & {\quad S < 0} \hfill \\ \end{array} } \right. $$

The surface’s derivative is provided by:

5 $$ \dot{S} = \frac{\partial S}{{\partial x^{T} }}\dot{x} = = \frac{\partial S}{{\partial x^{T} }}f(x) + \frac{\partial S}{{\partial x^{T} }}g(x)U_{eq} $$

6 $$ U_{eq} = - \frac{{\frac{\partial S}{{\partial x^{T} }}f\left( x \right)}}{{\frac{\partial S}{{\partial x^{T} }}g\left( x \right)}} $$

7 $$ \frac{\partial S}{{\partial x^{T} }}f(x) = \left( {\frac{{\partial^{2} i_{PV} }}{{\partial^{2} V_{PV} }}V_{PV} + 2\frac{{\partial i_{PV} }}{{\partial V_{PV} }}} \right)\frac{{i_{PV} }}{C} $$

8 $$ \frac{\partial S}{{\partial x^{T} }}g(x) = \left( {\frac{{\partial^{2} i_{PV} }}{{\partial^{2} V_{PV} }}V_{PV} + 2\frac{{\partial i_{PV} }}{{\partial V_{PV} }}} \right) \cdot - \frac{{i_{L} }}{C} $$

As a result, the equivalent control variable is depicted below.

9 $$ U_{eq} = \frac{{i_{PV} }}{{i_{L} }} $$

10 $$ U(t) = U_{eq} (t) + U_{n} (t) $$

11 $$ {\text{Where}}:\,U_{n} = Ksign\left( {s\left( x \right)} \right) $$

Nevertheless, the sign function causes chattering on the sliding surface, which is normally unfavorable^45,46^. Several strategies have been presented in the literature to decrease these oscillations, as may be stated^47^. In this paper, we suggest that the function sign be replaced with a function created via fuzzy logic. The universal command of the Law becomes:

12 $$ U(t) = U_{eq} (t) + U_{Fuzzy} (t) $$

#### MPPT controller with fuzzy logic

**Figure 6:**
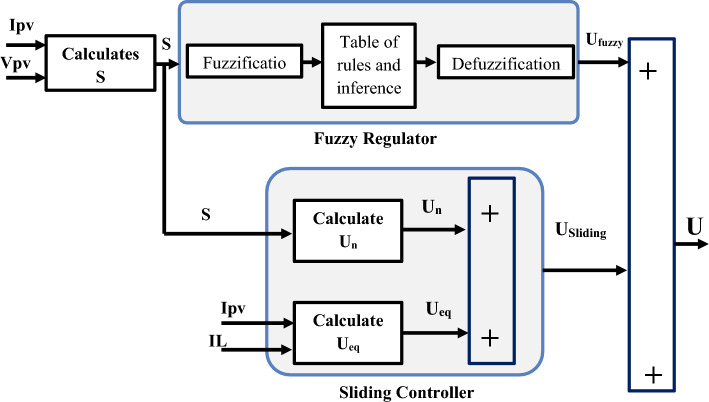
Structure of hybrid sliding fuzzy MPPT controller proposed.

Figure 6 depicts the suggested hybrid sliding fuzzy MPPT controller construction.

**Figure 7:**
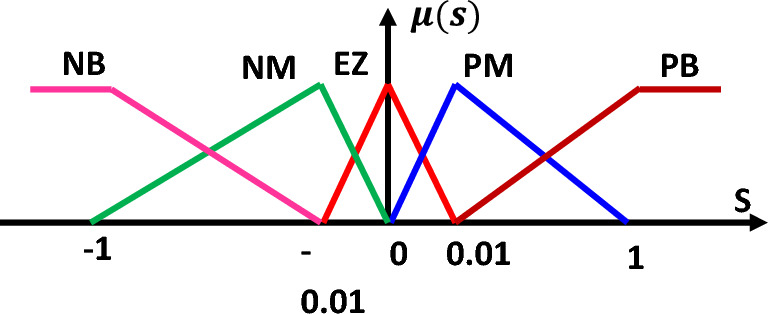
Input membership functions (S).

The following are the principal features of the fuzzy controller used^48,49^. As shown in Fig. 7, there are five fuzzy sets for the surface specified by triangle membership functions.

**Figure 8:**
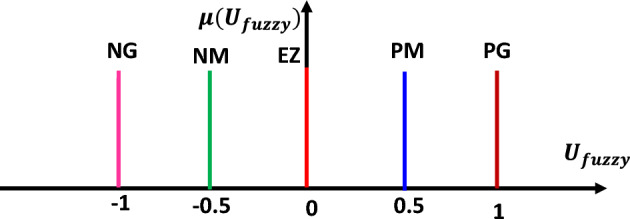
Output control membership functions (U_fuzzy_).

Figure 8 depicts the output control membership functions (Ufuzzy) using singletons membership forms.

Adoption of defuzzification based on the gravity center

13 $$ U_{{Fuzzy = \frac{{\mathop \sum \nolimits_{i = 1}^{5} {\varvec{\mu}}\left( {{\varvec{U}}_{{{\varvec{fuzzy}}}} } \right){\varvec{Ufuzzy}}}}{{\mathop \sum \nolimits_{i = 1}^{5} {\varvec{\mu}}\left( {{\varvec{U}}_{{{\varvec{fuzzy}}}} } \right)}}}} $$

#### Voltage of the bus

**Figure 9:**
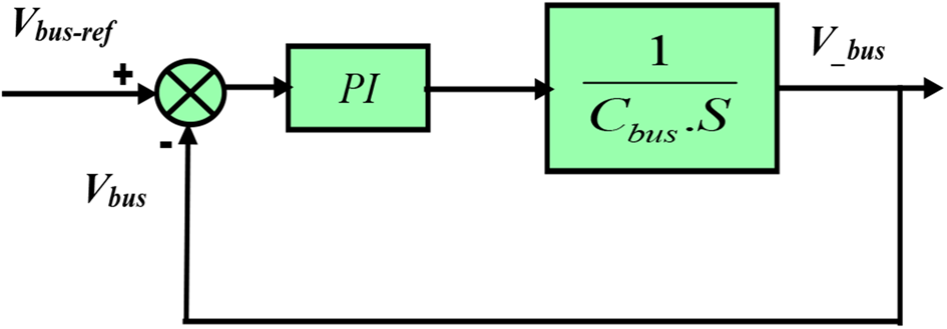
Control loop of the DC bus voltage.

A constant value is intended to be maintained via the DC bus voltage control (see Fig. 9).

The current of DC link is given by

14 $$ i_{C} = C \cdot \frac{{dV_{bus} }}{dt} = i_{L} - i_{bus} $$

Equation (16) has the following form in the Laplace domain:

15 $$ V_{bus} = \frac{{i_{C} }}{C \cdot S} $$

### Grid side management

#### Model of a voltage source inverter

The following statement relates the output voltages (Va, Vb, and Vc) and current of the inverter^50,51^:

16 $$ \left[ {\begin{array}{*{20}c} {{\text{V}}_{{\text{a}}} } \\ {{\text{V}}_{{\text{b}}} } \\ {{\text{V}}_{{\text{c}}} } \\ \end{array} } \right] = \frac{{{\text{V}}_{{\text{p}}} }}{3}\left[ {\begin{array}{*{20}c} 2 & {\quad - 1} & {\quad - 1} \\ { - 1} & {\quad 2} & {\quad - 1} \\ { - 1} & {\quad - 1} & {\quad 2} \\ \end{array} } \right]\left[ {\begin{array}{*{20}c} {{\text{K}}_{1} } \\ {{\text{K}}_{2} } \\ {{\text{K}}_{3} } \\ \end{array} } \right] $$

17 $$ {\text{I}}_{{{\text{inv}}}} = {\text{K}}_{1} {\text{I}}_{{\text{a}}} + {\text{K}}_{2} {\text{I}}_{{\text{b}}} + {\text{K}}_{3} {\text{I}}_{{\text{c}}} $$

#### PI Current control methodologies

The three-phase voltage of electrical grid is given by^52^:

18 $$ \left\{ {\begin{array}{*{20}l} {e_{a} = E_{m} \,\cos \,wt } \hfill \\ {e_{b} = E_{m} \,\cos \left( {wt - \frac{2n}{3}} \right)} \hfill \\ {e_{c} = E_{m} \,\cos \left( {wt + \frac{2n}{3}} \right)} \hfill \\ \end{array} } \right. $$

The voltage on the converter’s grid side employing Kirchhoff’s voltage laws may be expressed as law^53^:

19 $$ \left\{ {\begin{array}{*{20}l} {e_{a} = L\frac{d}{dt}i_{a} + Ri_{a} + v_{a} } \hfill \\ {e_{b} = L\frac{d}{dt}i_{b} + Ri_{b} + v_{b} } \hfill \\ {e_{c} = L\frac{d}{dt}i_{c} + Ri_{c} + v_{c} } \hfill \\ \end{array} } \right. $$

The Eq. (19) can be written as^54^:

20 $${e}_{abc}=L\frac{d}{dt}{i}_{abc}+R{i}_{abc}+{v}_{abc}$$

21 $$ \left( {\begin{array}{*{20}c} {\frac{{di_{a} }}{dt}} \\ {\frac{{di_{b} }}{dt}} \\ {\frac{{di_{c} }}{dt}} \\ \end{array} } \right) = \left( {\begin{array}{*{20}c} { - \frac{R}{L}} & {\quad 0} & {\quad 0} \\ 0 & {\quad - \frac{R}{L}} & {\quad 0} \\ 0 & {\quad 0} & {\quad - \frac{R}{L}} \\ \end{array} } \right)\left( {\begin{array}{*{20}c} {i_{a} } \\ {i_{b} } \\ {i_{c} } \\ \end{array} } \right) + \frac{1}{L}\left( {\begin{array}{*{20}c} {v_{a} - e_{a} } \\ {v_{b} - e_{b} } \\ {v_{c} - e_{c} } \\ \end{array} } \right) $$

From a three-phase ABC reference that was stationary to a two-phase DQ reference that was synchronously rotating.

22 $$ \left( {\begin{array}{*{20}c} {\frac{{di_{d} }}{dt}} \\ {\frac{{di_{q} }}{dt}} \\ \end{array} } \right) = \frac{1}{L}\left( {\begin{array}{*{20}c} { - R} & {wL} \\ {wL} & { - R} \\ \end{array} } \right)\left( {\begin{array}{*{20}c} {i_{d} } \\ {i_{q} } \\ \end{array} } \right) - \frac{1}{L}\left( {\begin{array}{*{20}c} {e_{d} } \\ {e_{q} } \\ \end{array} } \right) + \frac{1}{L}\left( {\begin{array}{*{20}c} {v_{d} } \\ {v_{q} } \\ \end{array} } \right) $$

Equation (22) can be simplified as follow^55^

23 $$ \left\{ {\begin{array}{*{20}l} {e_{d} = L\frac{d}{dt}i_{d} - wLi_{q} + v_{d} + Ri_{d} } \hfill \\ {e_{q} = L\frac{d}{dt}i_{q} + wLi_{d} + v_{q} + Ri_{q} } \hfill \\ \end{array} } \right. $$

The following are the control equations^56^:

24 $$ \left\{ {\begin{array}{*{20}l} {v_{d} = \left( {K_{p} + \frac{{K_{i} }}{S}} \right)\left( {i_{dref} - I_{d} } \right) - wLi_{q} + e_{d} } \hfill \\ {v_{q} = \left( {K_{p} + \frac{{K_{i} }}{S}} \right)\left( {i_{qref} - I_{q} } \right) + wLi_{d} + e_{q} } \hfill \\ \end{array} } \right. $$

where K_p_ and K_i_ are the PI current controllers gains, respectively.

#### Tuning of PI current control methodologies

The output of a PI regulator is given by:

25 $$ u\left( t \right) = Kp \cdot e\left( t \right) + \frac{Ki}{{Ti}}\int_{0}^{t} {e\left( t \right)dt} $$

26 $$ e\left( t \right) = Irefdq - Idq $$

##### Tuning of PI current control using PSO approaches

Kennedy and Eberhart initially recommended the PSO method in 1995. This contemporary heuristic approach is based on the behavior and intelligence of swarms^57–60^.

Tuning a Proportional-Integral (PI) controller for current control using Particle Swarm Optimization (PSO) involves finding the optimal values for the proportional gain (K_P_) and integral gain (K_I_) parameters to achieve desired control performance. Here’s a step-by-step explanation of how this process can be carried out:

Define objective function:

The objective function represents the performance criteria of the control system. In this case, it could be minimizing steady-state error, achieving a desired response time.

The Integrated of Squared Error (ISE) is defined by:

$$ {\text{ISE}} = \int_{0}^{\infty } {{\text{e}}^{2} {\text{dt}}}  $$

The objective function (F) isdetermined), according to the following criteria:

27 $$F=\sum_{k=1}^{N}{\left[Idref\left(k\right)-Id\left(k\right)\right]}^{2}$$

where N is the amount of iterations, id is the direct current components, and id_ref_ is the reference direct current components received from the PV array.

Parameter initialization:

- Initialize the population of particles. Each particle represents a potential solution, consisting of K_P_ and K_i_ values.
- Randomly initialize the position and velocity of each particle within a defined search space.
- Define the inertia weight (w), acceleration coefficients (c1 and c2), and maximum velocity limits.

Evaluate fitness

- Evaluate the fitness of each particle by calculating the objective function based on its position (K_P_, K_i_).

Update personal and global bests

- Update the personal best position (pbest) for each particle if its current fitness is better than its previous best.
- Update the global best position (gbest) if any particle has found a better solution compared to the previous global best.

Update particle velocities and positions

- Update the velocity of each particle using the following equation^61–63^
  28 $$ \begin{aligned} v_{i,j}^{{\left( {k + 1} \right)}} & = w \times v_{i,j}^{\left( k \right)} + c_{1} \times rand\left( x \right) \times \left( {pbest_{i,j} - x_{i,j}^{\left( k \right)} } \right) + c_{2} \times rand\left( x \right) \times \left( {gbest_{i,j} - x_{i,j}^{\left( k \right)} } \right) \\ x_{i,j}^{{\left( {k + 1} \right)}} & = x_{i,j}^{\left( k \right)} + v_{i,j}^{{\left( {k + 1} \right)}} \\ \end{aligned} $$
  where *i* is the number of individuals in a group, *j* is the PI parameter number, *x* is the PI parameter, $v$ is the velocity, $pbest$ is the personal best of individual *i,*$gbest$ is a global best of all individuals,$w,{C}_{1}$ and ${C}_{2}$ are weight parameters ,$rand\left(x\right)$ is a uniform random number from 0 to 1.

##### Tuning of PI current control using a genetic algorithm (GA)

**Figure 10:**
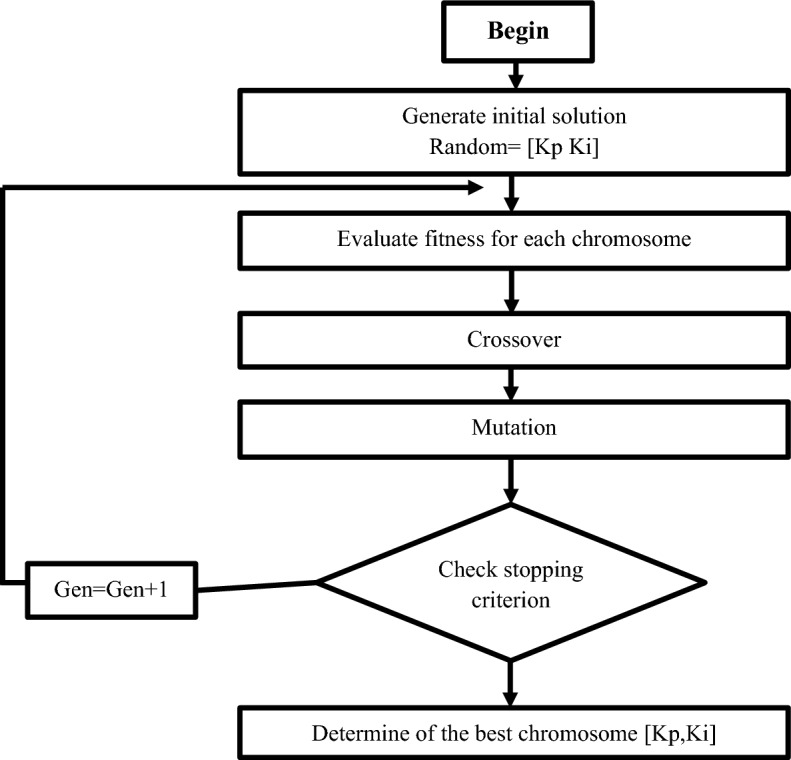
A flowchart for tuning the proportional gain (K_p_) and integral gain (K_i_) of a PI controller using a genetic algorithm.

The parallelism observed in nature is used by GA algorithm, a smart optimization approach. Specifically, its search techniques are based on the principles of natural choice and genetics. Holland is credited with creating the genetic algorithm in the early 1970 s^64^. GA operates on a population, which is a grouping a number potential solutions. Each individual or solution is referred to as a chromosome, and each unique character is referred to as a gene. Each iteration involves the evolution of a new generation in order to provide better solutions (population) than the previous one^65^. The percentage of people in the solution who are substituted from one generation to the next as the generation gap^66^. To achieve optimal control performance under nominal operating conditions, GA can be used to tune PI position controller gains. Below is a basic flowchart illustrating the main stages of this procedure (see Fig. 10):

The Genetic Algorithm Toolbox (GATool) in MATLAB to tune the proportional gain (Kp) and integral gain (Ki) of a PI controller.

**Figure 11:**
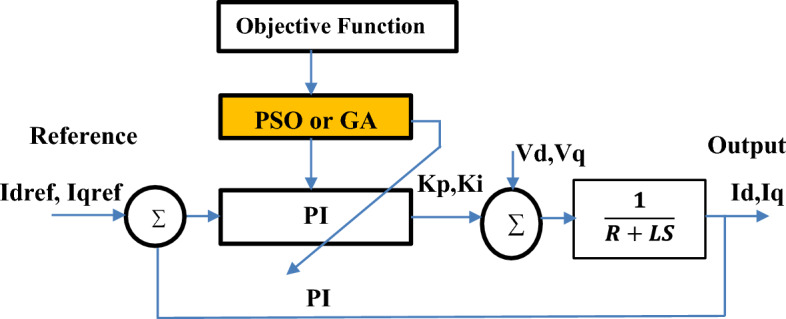
Tuning a Proportional-Integral (PI) current controller using Particle Swarm Optimization (PSO) and Genetic Algorithms (GA).

Figure 11 illustrates the process diagram of the PI parameter tuning of the PI current regulators in the grid side based on both PSO and GA approaches. The goal is to find the optimum gain of the PI current controller (K_p_ and K_i_).

Both PSO and GA offer effective methods for tuning the parameters of a PI current controller. PSO tends to be faster and simpler to implement, while GA provides a more systematic exploration of the search space^67^. The choice between PSO and GA may depend on factors such as the complexity of the control system, desired optimization performance, and computational resources available.

## Simulation results

A simulation model of plants is built using the MATLAB tool to examine the efficacy of the suggested control measures. 16 PV modules (S235P60 Centro Solar S-Class Professional Polycrystalline), a static boost converter, and a grid-connected inverter make up the system. The line resistance and output filter inductances are both 0.1 ohms and 3 mH, respectively. The suggested hybrid sliding fuzzy MPPT technique is evaluated in the southern Algerian city of Ghardaia under two distinct irradiation profiles: sudden variation and real irradiation profile.

### A sudden variation of irradiation

**Figure 12:**
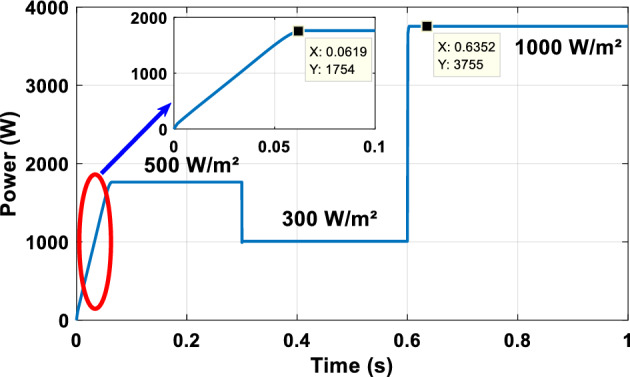
PV array power output.

The output of the PV array under sudden change in irradiation is illustrated in Fig. 12, G = 500 W/m^2^, from t = 0 to 0.3 s, from t = 0.3 to 0.6 s, G = 300 W/m^2^, and from t = 0.6 to 1 s, G = 1000 W/m^2^.

### Real solar irradiation profile

In this section, the grid-tied PV system is subjected to an actual radiation profile of Ghardaia (Algeria) for four days (from April 22 to April 25). Irradiation grew daily from 0 to 1000 W/m^2^, from 6:00 a.m. to 6:00 p.m., and there was none at night while the temperature was stable at 25 °C. The optimal PI controller gains are provided in Tables 2, and Table 1 defines the PSO factors for generating an initial random population of people corresponding the PI controller gains (K_p_ and K_i_). Table 2 lists the optimum PI controller gains.

**Table 1:** PSO factors.

Descriptions	Values
Population size	20
Maximum iteration	20
*c*1 = *c*2	2

**Table 2:** PSO parameters.

Gains	Value
K_p_	9
K_i_	300

We used Gatool from the MATLAB toolbox to run the GA algorithm, with the following settings: Normalized geometric selection for selection, Arithmetic crossover for crossover, and a uniform approach for mutation. Table 3 lists the parameters of the genetic algorithm that were selected for tuning.

**Table 3:** GA algorithm values.

GA parameters	Value/Method
Solution dimension	60
Variable bounds [K_p_ K_i_]	[0 400;0 400]
Most generations possible	100
Selection process	Normalized geometric selection
Crossover process	Arithmetic crossover
Mutation process	Uniform mutation
Performance index/Fitness function	Mean square error MSE

### The photovoltaic array chosen

**Figure 13:**
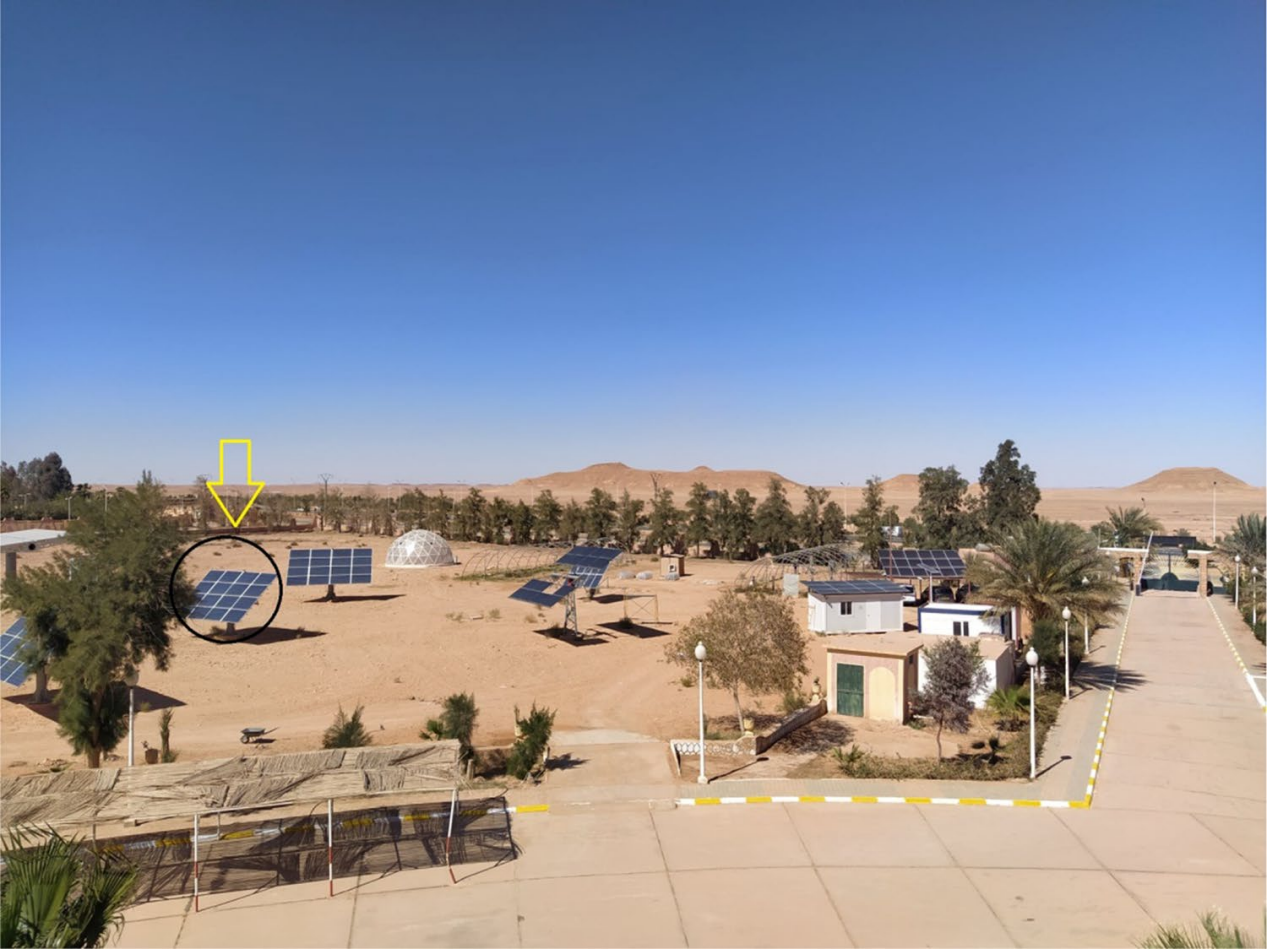
A photo of the investigated PV array.

The solar array that was chosen for this study is situated in Ghardaia, Algeria, in an applied research project on renewable energy. It is made up of 16 S235P60 Centro Solar S-Class Professional Polycrystalline PV Modules. A photo of the investigated PV field is depicted in Fig. 13. Tables 4 and 5 lists the features of the PV arrays.

**Table 4:** PV module specifications.

S235P60 centro solar S-class professional polycrystalline PV module	
Power Pmpp	235 Wc
Isc	8.59 A
Uoc	36.46 V
Voltage (Umpp)	28.70 V
Current (Impp)	8.19 A
Module efficiency ɳ	14.3%

**Table 5:** PV field Electrical spesification at STC.

Power Pmpp	3.76 kWp
Uoc	546.9 (V)
Isc	8.59 (A)
Voltage Vmp	459.2 (V)
Current Imp	8.19 (A)
Module number	16

**Figure 14:**
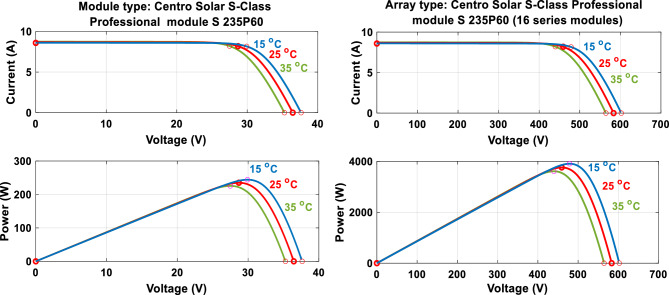
I (V) and P(V) specification of the solar panel and the PV array.

The influence of temperature on the behavior of the solar panel and the PV field is depicted in Fig. 14.

**Figure 15:**
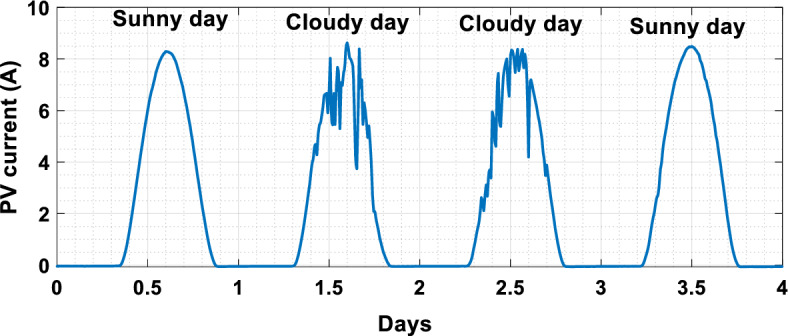
Daily evolution of PV current within four days.

**Figure 16:**
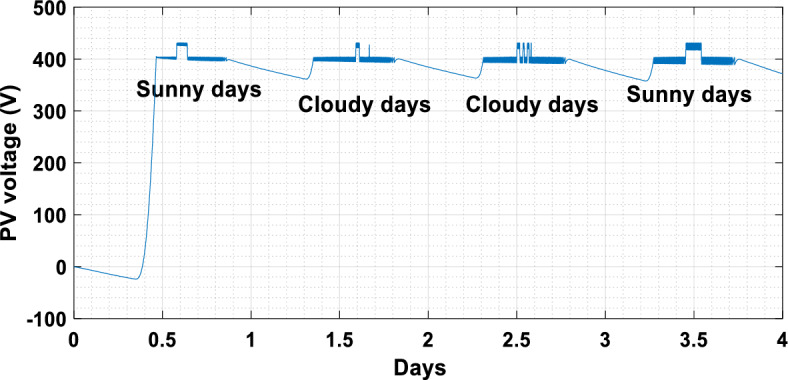
The daily evolution of PV voltage within four days.

**Figure 17:**
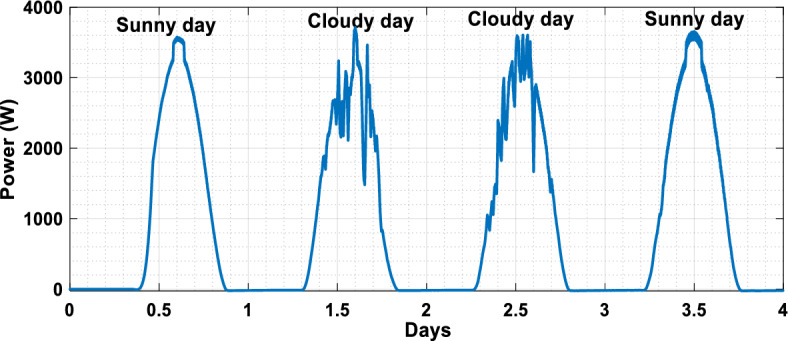
The daily evolution of PV power within four days.

**Figure 18:**
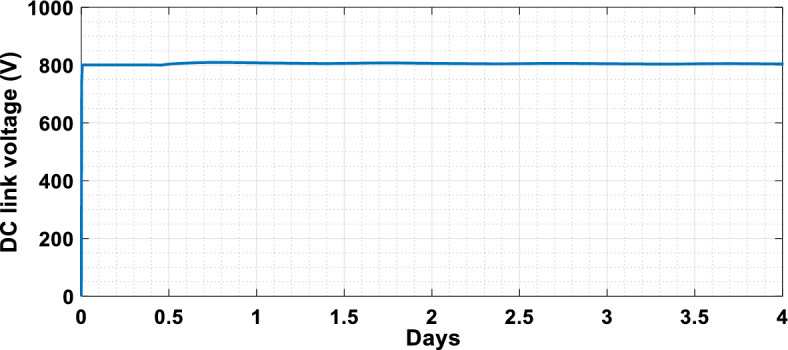
DC link Voltage profile within four days (from 22/04/2015 to 25/04/2015).

**Figure 19:**
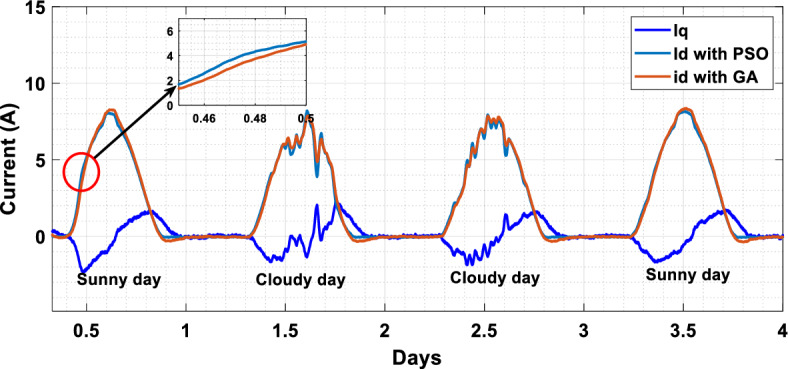
Output current Id and Iq within four days (from 22/04/2015 to 25/04/2015).

**Figure 20:**
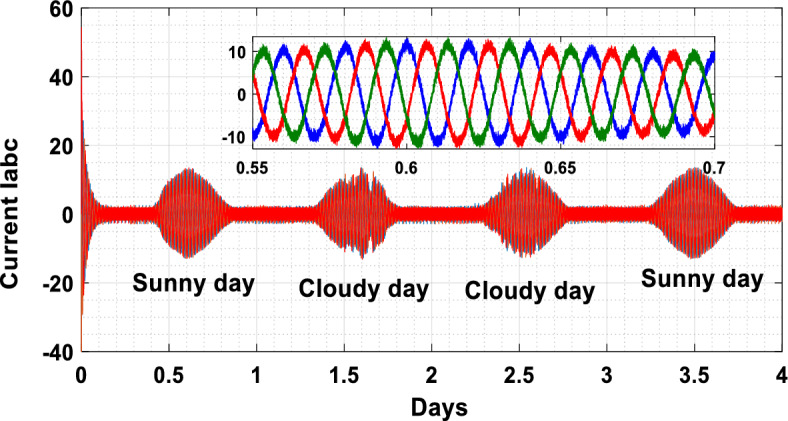
Three phase output current Iabc within four days.

**Figure 21:**
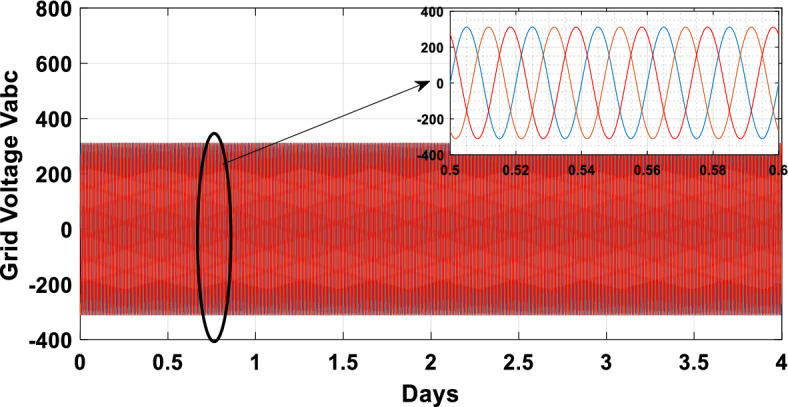
Three phase grid voltages profile within four days.

**Figure 22:**
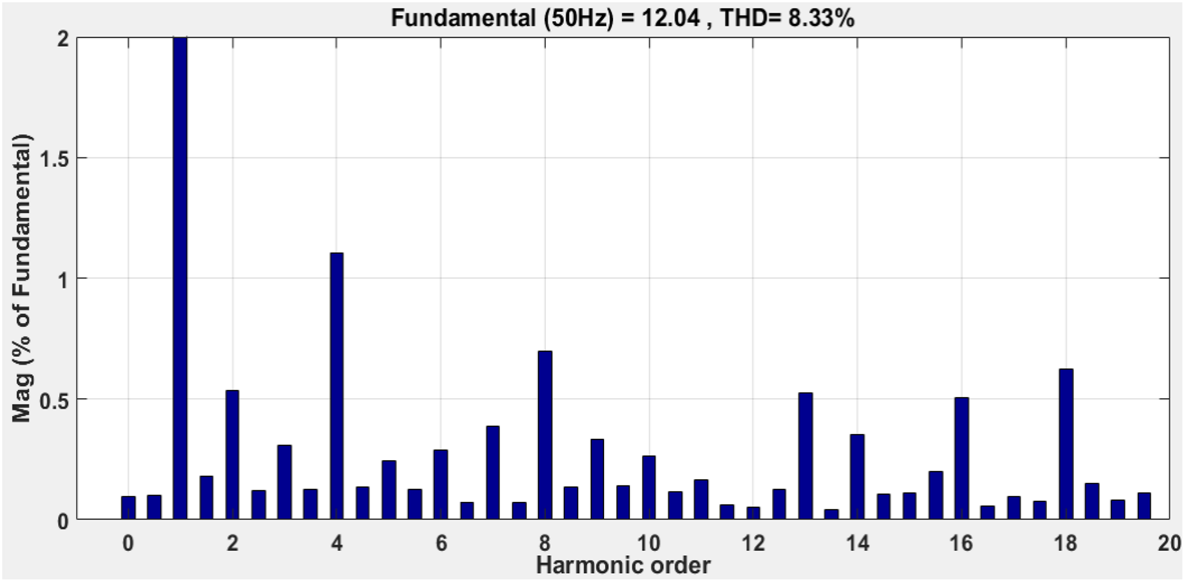
Total harmonic distortion (THD) of the Current applying PSO method.

**Figure 23:**
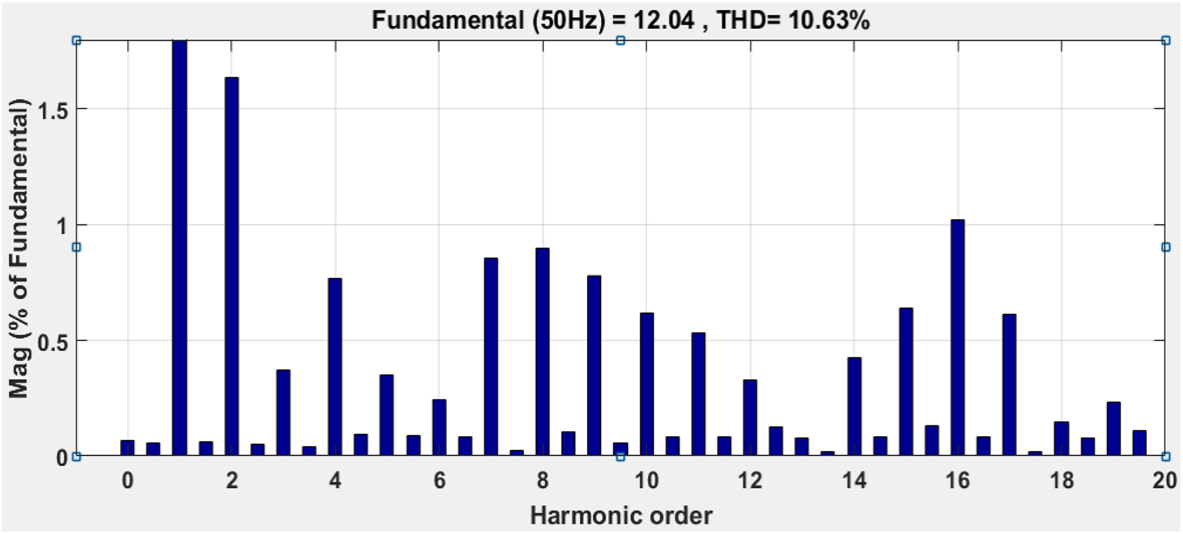
Total harmonic distortion (THD) of the Current applying GA algorithm.

**Figure 24:**
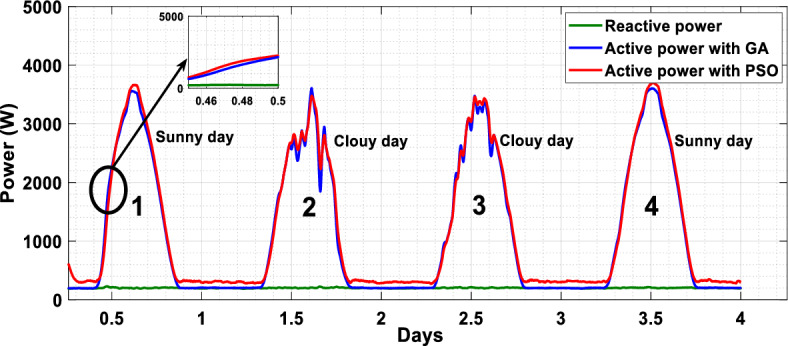
Active and reactive power profile within of four days.

The suggested technique has been evaluated in a variety of weather scenarios (two days with sunshine and two days with clouds). The tracking of the MPP and the inverter control exhibit great performance, good robustness, and quick reactions, according to the results. Figures 15, 16, and 17 display the PV panel’s results for the four days from April 22–25, 2015. As can be seen, after an adequate response time of t = 0.01 s with relation to the gradual variations of the input source profile of radiation and temperatures, the power, current, and voltage control features are all in good agreement with their references. The current is increasing up to 8.19 A, while the PV voltage is approximately constant. The voltage bus U_DC_ is regulated to their reference 800 V, as appearing in Fig. 18. The waveforms of the injected current to the network are appearing in Fig. 19 based on PSO and Genetic GA algorithms to determine the best regulator settings. The three phases current may be observed to closely resemble the reference. The electrical voltage and current on the network-side lines are in the same phase, as can be seen in these figures. They are sinusoidal and in phase. A unit power factor is attained as a consequence. Figures 20 and 21 display both the voltage and electrical current waveforms of the network in three phases. It is presumed that the conventional network voltage has a stable amplitude and frequency. The THD is examined in Figs. 22, 23, the measurement of the current waveform is “distorted” or changed by about 8.33% using the PSO algorithm (see Fig. 22). The measurement of the current waveform is “distorted” or changed by about 10.63% using the GA algorithm (see Fig. 23). Figure 24 shows how much power is active and reactive supplied to the network over a period of four days in relation to solar irradiation. Table 6 shows a performance comparison between hybrid sliding fuzzy and other MPPT methods.

**Table 6:** Performance comparison between hybrid sliding fuzzy and other MPPT methods.

Methods	MPP of P–V curve (W)	Extracted PV power (W)	Power loss		Efficiency (%)	Convergence time (S)	Reference
			(W)	(%)			
Hybrid sliding fuzzy	3760	3755	5	0.13	99.86	0.06	Present study
CSA	325.5	325.40	0.1	0.03	99.97	0.31	^68^
P&O	325.5	323.10	2.4	0.74	99.26	0.65	^68^
PSO	1260	1244	16	1.27	98.7	0.47	^69^
InC	864	863.1	0.9	0.10	99.90	0.52	^70^
Firefly algorithm (FA)	864	852	12	1.39	98.61	0.60	^70^
Ant colony optimization (ACO)	864	862.2	1.8	0.21	99.76	0.53	^70^
Dragonfly optimization (DFO)	864	863.3	0.7	0.08	99.77	0.53	^70^

A detailed comparison with previous research work is presented in Table 7 with specific parameters (speed of tracking, accuracy, and efficiency).

**Table 7:** Comparison of fuzzy sliding MPPT outcomes with those of different MPPT methods.

	Speed of tracking	Accuracy	Efficiency	Complexity	Reference
Fuzzy sliding	Very fast	Accurate	99.86	Simple	Present study
P&O	Slow	Low	94.96	Simple	^71,72^
InC	Slow	Medium	95.60	Simple	^71^
SMC	Fast	High	> 98%	Med	^73^
ANN	Medium	Accurate	97.84	Medium	^71^
FLC	Medium	Accurate	96.88	Medium	^37,71^
ANFIS	Medium	Accurate	97.76	Medium	^71^
ACO	Fast	High	> 98.5%	Low	^73^
GA	Med	Med	> 98%	Low	^73^
PSO	Med	Med	> 98%	Low	^74^
ABC	Fast	High	> 99%	Med	^73^
FA	Fast	High	> 98.5%	Medium–high	^73^
GWO	Medium	High	–	Simple	^75^
PSO-P&O	High	Medium	100%	Medium to complex	^75^
FLC-P&O	Fast	High	High	Simple	^76^
ACO-P&O	Faster	High	High	Simple	^76^
ANN-P&O	Fast	High	High	Simple	^76^
GWO-P&O	Medium	High	99.77%	Medium	^76^

## Conclusion and future research directions

The novel hybrid Maximum Power Point Tracking (MPPT) technique, combining fuzzy logic and sliding mode control, presents a promising and innovative solution for enhancing the overall performance of grid connected Photovoltaic (PV) systems operating in variable and real atmospheric conditions (case study of Ghardaia). Using intelligent techniques (PSO and GA), the PI parameters of a grid-tied PV system control were tuned on the grid side to find the best possible gains. The simulation has been conducted utilizing the Matlab Simulink package. The outcomes of the proposed controller show better performance (speed of tracking, accuracy, and efficiency) and are very satisfactory which demonstrates their effectiveness. The suggested y MPPT technique delivers a considerable improvement in tracking efficiency of 99.86%, a time response of 0.06 s, and less oscillation than the previous methods. According to considered IEEE standards for low-voltage networks, the total current harmonic distortion values (THD) obtained are considerably high (8.33% and 10.63% using, PSO and GA algorithms respectively). Simulation results have substantiated the superior performance of the hybrid MPPT technique when compared to traditional methods. The hybrid approach consistently demonstrated improved tracking efficiency, faster response times, and enhanced stability under varying atmospheric conditions. These findings underscore the potential of the proposed technique to significantly elevate the energy yield and reliability of PV systems, making it a viable and attractive option for real-world applications in renewable energy.

In considering future research directions stemming from this study, several key areas emerge for further investigation. Firstly, there is a pressing need to delve deeper into strategies for mitigating Total Harmonic Distortion (THD) in current waveforms within grid-connected PV systems. This could involve the development and refinement of advanced control algorithms, such as adaptive or predictive techniques, aimed at minimizing THD levels while optimizing system efficiency. Additionally, exploring the integration of energy storage solutions, such as batteries or supercapacitors, into grid-connected PV systems presents a promising avenue for enhancing system stability and reliability, particularly in regions prone to fluctuations in solar irradiation. Furthermore, expanding the scope of analysis to encompass a wider range of environmental variables, including cloud cover, humidity, and wind speed, would provide valuable insights into the performance of the proposed hybrid MPPT technique under diverse climatic conditions. Moreover, investigating the feasibility of deploying distributed PV systems within smart grid frameworks could offer new opportunities for improving energy management and grid stability at the local level. Lastly, the development of advanced predictive modeling frameworks leveraging machine learning algorithms, such as neural networks or support vector machines, holds potential for enhancing the accuracy of solar irradiation forecasting, thereby enabling more proactive and adaptive control strategies for grid-connected PV systems. By addressing these research challenges, the field of renewable energy stands to benefit from enhanced system performance, reliability, and integration within the broader energy landscape.

## Data Availability

The datasets used and/or analysed during the current study available from the corresponding author on reasonable request.
