# Author Correction: Enhancing grid‑connected photovoltaic system performance with novel hybrid MPPT technique in variable atmospheric conditions

**DOI:** 10.1038/s41598-024-66134-6

**Published:** 2024-07-02

**Authors:** Layachi Zaghba, Abdelhalim Borni, Messaouda Khennane Benbitour, Amor Fezzani, Abdullah Alwabli, Mohit Bajaj, Shir Ahmad Dost Mohammadi, Sherif S. M. Ghoneim

**Affiliations:** 1 Centre de Développement des Energies Renouvelables, CDER, Unité de Recherche Appliquée en Energies Renouvelables, URAER, 47133 Ghardaïa, Algeria; 2 Department of Electrical Engineering, College of Engineering and Computing in Al-Qunfudhah, Umm Al-Qura University, Mecca, Saudi Arabia; 3 Department of Electrical Engineering, Graphic Era (Deemed to Be University), Dehradun, 248002 India; 4 Hourani Center for Applied Scientific Research, Al-Ahliyya Amman University, Amman, Jordan; 5 Graphic Era Hill University, Dehradun, 248002 India; 6 Applied Science Research Center, Applied Science Private University, Amman, 11937 Jordan; 7 Department of Electrical and Electronics, Faculty of Engineering, Alberoni University, Kapisa, Afghanistan; 8 Department of Electrical Engineering, College of Engineering, Taif University, 21944 Taif, Saudi Arabia

Correction to: *Scientific Reports* 10.1038/s41598-024-59024-4, published online 08 April 2024

The original version of this Article contained an error in the spelling of the author Messaouda Khennane Benbitour which was incorrectly given as Messaouda Khennane Benbiotur.

The original Article has been corrected.

